# Efficacy of Acupuncture Combined With Pharmacotherapy for Depression: A Randomized Controlled Clinical Trial

**DOI:** 10.31083/AP52350

**Published:** 2026-08-13

**Authors:** Shuyun Huang, Sumiao Zhou, Lida Zhang, Fengchun Wu, Zhendong Zhang, Yuping Ning, Zixuan Liu, Jingping Wu, Bingxin Chen, Qiuling Lu, Li Gao, Zhimin Zhu, Wei Han, Yuanyuan Huang

**Affiliations:** ^1^Department of Psychiatry, The Affiliated Brain Hospital, Guangzhou Medical University, 510370 Guangzhou, Guangdong, China; ^2^Guangdong Engineering Technology Research Center for Translational Medicine of Mental Disorders, 510370 Guangzhou, Guangdong, China

**Keywords:** acupuncture, depression, treatment efficacy, anxiety, selective serotonin reuptake inhibitors

## Abstract

**Background:**

Depression is frequently accompanied by comorbid symptoms such as anxiety, insomnia, and cognitive impairment, which complicate treatment and adversely affect clinical outcomes. Acupuncture has shown promise as a complementary therapy; however, the efficacy of acupuncture combined with selective serotonin reuptake inhibitors (SSRIs) requires further investigation.

**Methods:**

This single-center, randomized clinical trial enrolled 213 inpatients with depression and comorbid symptoms. Participants were randomly assigned to receive either SSRIs treatment or antidepressant treatment combined with acupuncture for two weeks. Clinical outcomes were assessed using the Hamilton Depression Rating Scale (HAMD), Hamilton Anxiety Rating Scale (HAMA), Mini-Mental State Examination (MMSE), Pittsburgh Sleep Quality Index (PSQI), and Beck Scale for Suicide Ideation (BSI). The primary outcome was the change in the HAMD reduction rate.

**Results:**

Both groups demonstrated significant clinical improvement after two weeks of treatment. However, analysis of covariance (ANCOVA) revealed that the acupuncture group achieved significantly greater reductions in HAMD, HAMA, and PSQI scores, as well as significantly higher MMSE scores, compared with the control group (all adjusted *p* < 0.05). Hierarchical regression analyses indicated that baseline symptom severity and duration of illness were significant predictors of treatment response.

**Conclusions:**

When used as an adjunct to antidepressant therapy, acupuncture provides rapid and broad therapeutic benefits for patients with depression. Compared with SSRI monotherapy, the combined treatment was associated with greater improvements in depressive and anxiety symptoms, sleep quality, and cognitive performance. These findings support the integration of acupuncture into multimodal treatment strategies for managing the complex symptom profile of major depressive disorder.

**Clinical Trial Registration:**

The study has been registered on https://itmctr.ccebtcm.org.cn/en/index.html (registration number: ITMCTR2025001802; registration link: https://trialsearch.who.int/Trial2.aspx?TrialID=ITMCTR2025001802).

## Main Points

1. Acupuncture combined with selective serotonin reuptake inhibitors (SSRIs) significantly improved depressive symptoms and common comorbid symptoms in patients with depression after two weeks of treatment.

2. Combined treatment demonstrated superior efficacy compared with pharmacotherapy alone, particularly in reducing depressive and anxiety symptoms.

3. Patients receiving adjunctive acupuncture showed greater improvements in sleep quality and cognitive function than those receiving pharmacotherapy alone.

4. Baseline depression severity, anxiety severity, and duration of illness were significant predictors of treatment response, highlighting the potential value of individualized treatment strategies.

## 1. Introduction

Depression is a common mental disorder, affecting over 350 million people worldwide. Its prevalence has increased, by 18% increase in prevalence over the past decade according to the World Health Organization [1]. In China alone, more than 95 million individuals are estimated to suffer from depression, with a lifetime prevalence of 6.9% and a 12-month prevalence of 3.6% [2]. Typical symptoms include persistently low mood and anhedonia, often accompanied by suicidal ideation, anxiety, or somatic complaints such as cardiovascular symptoms [3]. Although pharmacotherapy remains the most widely used first-line treatment (particularly selective serotonin reuptake inhibitors [SSRIs]), it presents several drawbacks, including delayed onset, adverse effects, and high interindividual variability in therapeutic response. Notably, only about 60% of patients benefit from SSRIs [4], and approximately 30%–40% of adolescents exhibit poor responses to antidepressants [5]. These limitations highlight the need for more effective and comprehensive treatment strategies. Recent meta-analyses suggest that combined therapies integrating pharmacological and non-pharmacological interventions may outperform monotherapies in improving treatment response and overall functional outcomes [6,7].

Acupuncture is a widely accepted complementary therapy and has demonstrated effectiveness in treating depressive symptoms due to its convenience, minimal adverse effects, and broad clinical acceptance [8]. As a key component of Traditional Chinese Medicine (TCM), it is based on the principle of stimulating specific peripheral points (acupoints) to regulate the flow of “*Qi*” and restore homeostatic balance. From a modern physiological perspective, acupuncture is increasingly recognized as a form of peripheral sensory stimulation that modulates the central nervous system. This process, often referred to as “acupuncture neuromodulation”, involves the activation of high-threshold sensory fibers that transmit signals to the spinal cord and brain, subsequently influencing the release of various neurotransmitters and neuropeptides such as endorphins, serotonin, and dopamine. Mechanistic studies indicate that acupuncture modulates neurobiological pathways, including anti-inflammatory and antioxidant effects, neuroplasticity enhancement, and the regulation of neurotransmitters and immune function [9,10]. A recent clinical trial reported that after 6 weeks of treatment, patients receiving acupuncture combined with fluoxetine exhibited decreased levels of TNF-α, IL-6, and IL-1β, increased BDNF levels, and reduced miR-155 expression, indicating that acupuncture may help to restore immune balance by reducing inflammation [11]. Previous studies have suggested that acupuncture may modulate inflammatory responses through neuroimmune pathways, including regulation of immune activity and inflammatory mediators [12]. Endorphins have been shown to suppress macrophages, natural killer cells, and T cells, thereby reducing neuroinflammation and pain [13]. It has been proposed that acupuncture increases endorphin expression, which, through μ-opioid receptors, suppresses inflammation and ameliorates depressive symptoms [14]. Depression is broadly associated with neuroinflammation and blood-brain barrier disruption, leading to neuronal loss, monoamine deficiency, and impaired brain network connectivity. Acupuncture may alleviate symptoms by enhancing neuroplasticity and reducing inflammation [9]. Animal research has provided preliminary support for its antidepressant effects, such as alleviating depressive-like behaviors in rodent models via stimulation at Baihui and Yintang [15,16]. Abdominal and electroacupuncture modalities have also demonstrated activation of reward circuits and modulation of central signaling pathways [17]. However, most clinical studies to date have used standardized or limited acupoint combinations, resulting in insufficient methodological innovation in acupuncture protocols. Moreover, reviews and meta-analyses dominate the current literature, with a relative paucity of large-scale, well-controlled clinical trials [18,19]. The lack of direct comparisons between acupuncture and pharmacotherapy, especially using novel acupuncture techniques, remains a significant gap in knowledge.

Depression frequently coexists with comorbid symptoms such as anxiety, insomnia, and cognitive dysfunction, all of which contribute to poorer functional outcomes. Meta-analytic evidence indicates that the efficacy of acupuncture is not necessarily dependent on whether it is combined with pharmacotherapy [20]. A systematic review comparing acupuncture to antidepressants and sham acupuncture found that acupuncture produced fewer adverse effects and significantly improved Hamilton Depression Rating Scale (HAMD) scores [21]. However, few studies have directly compared the effectiveness of combined acupuncture and medication with medication alone, particularly in relation to comorbid symptoms. Anxiety is one of the most prevalent comorbidities in depression, exacerbating functional impairment [22], and acupuncture has demonstrated independent efficacy in treating anxiety [23]. Similarly, insomnia is highly prevalent among patients with depression, but conflicting findings exist regarding the impact of acupuncture on sleep quality [24,25]. Cognitive impairment is another common issue in depression, with preliminary evidence supporting the benefit from acupuncture [26]. Despite these observations, most studies to date have only addressed depression or its comorbidities in isolation, often in small samples and without standardized protocols. Hence, there is a lack of integrative, high-quality studies that target both core and comorbid symptoms in a combined treatment framework.

In light of these limitations, the aim of the present study was to evaluate the efficacy of acupuncture incorporating specific acupoints such as Dazhui and Fengfu. Although these two acupoints have shown therapeutic potential in preliminary studies [27], previous research has mainly focused on acupuncture combined with pharmacotherapy, whereas the efficacy of this acupuncture approach alone and its comparative effectiveness against pharmacotherapy remain insufficiently investigated. To address this gap, we conducted a randomized controlled trial (RCT) to compare the efficacy of acupuncture combined with antidepressants versus antidepressants alone in 213 patients with depression and comorbid symptoms. We hypothesized that combined treatment has superior clinical outcomes, particularly in alleviating depressive and comorbid symptoms. Additionally, we explored potential baseline predictors of treatment response to inform individualized treatment strategies. Overall, our aim was to provide new clinical evidence supporting the use of integrative therapies in managing depression with complex symptom presentations.

## 2. Materials and Methods

### 2.1 Study Design

**Fig. 1.**
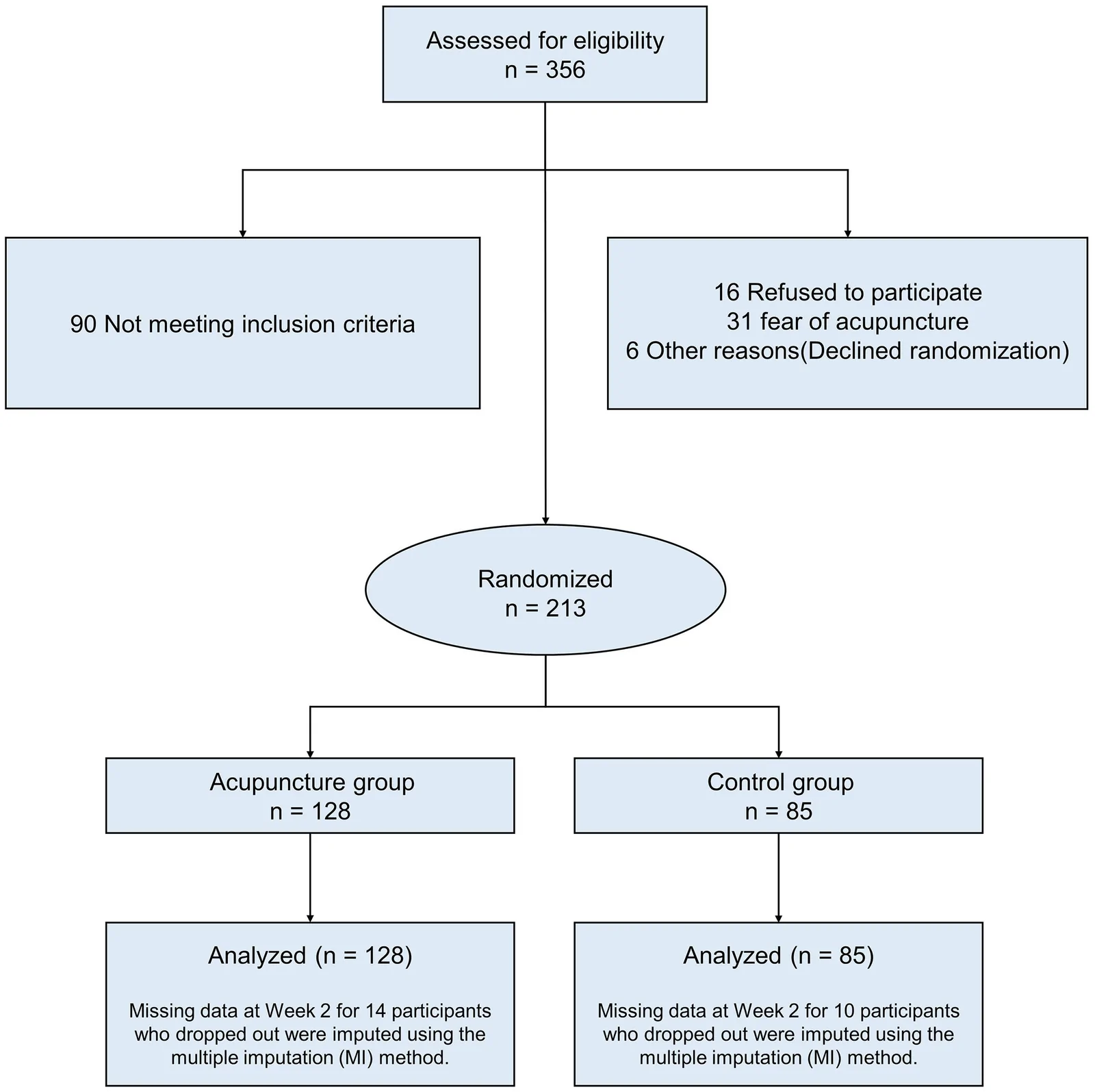
**CONSORT flow diagram showing participant recruitment, randomization, follow-up, and analysis, the dropout rate was 10.9% in the acupuncture group and 11.7% in the control group, showing no significant difference between groups (*****p >***** 0.05)**. CONSORT, Consolidated Standards of Reporting Trials.

This single-center RCT was approved by the Ethics Committee of the Affiliated Brain Hospital, Guangzhou Medical University in China. It was conducted from October 1, 2025, to February 28, 2026, with participants recruited from the Brain-Gut Health Initiative (BIGHI) cohort, a large-scale prospective study designed to investigate the multi-dimensional signatures of psychiatric disorders in the Chinese population [28]. To evaluate the efficacy of acupuncture combined with SSRIs for the treatment of depression, we conducted a superiority trial comparing the combined treatment (acupuncture + SSRIs) with pharmacotherapy alone (SSRIs). The primary outcome was the percentage reduction in total Hamilton Depression Rating Scale (HAMD) score after 2 weeks of treatment. A total of 356 participants were screened for eligibility, of whom 143 were excluded for not meeting the inclusion criteria. The remaining 213 participants were randomly assigned to the combined treatment group (n = 128) and the pharmacotherapy alone group (n = 85) in a 3:2 ratio. During the study period, 24 participants dropped out due to withdrawal of informed consent, revision of diagnosis during hospitalization, or poor compliance (14 in the combined treatment group and 10 in the pharmacotherapy alone group). Based on the intention-to-treat (ITT) principle, all 213 randomized participants (128 in the combined treatment group and 85 in the pharmacotherapy alone group) were included in the final efficacy analyses. Missing primary and secondary outcome data at Week 2 for these participants were handled using the multiple imputation (MI) method based on a fully conditional specification. The trial procedure is outlined in Fig. 1. Patients or the public were not involved in the design, conduct, or reporting of this research. The study followed reporting guidelines from the Consolidated Standards of Reporting Trials (CONSORT). No changes were made to the protocol commencement of the trial, and no interim analyses or stopping rules were planned.

### 2.2 Participants

All participants provided written informed consent after a thorough explanation of the study procedures.

Inclusion criteria for both groups were: (1) patients aged between 18 and 60 years; (2) diagnosis of major depressive episode based on the Fifth Edition of the Diagnostic and Statistical Manual of Mental Disorders (DSM-5); (3) 17-item Hamilton Depression Rating Scale (HAMD-17) score ≥17 and Young Mania Rating Scale (YMRS) scores ≤5; and (4) at least 6 years of education, with the ability to understand the study protocols and provide written informed consent.

Exclusion criteria included: (1) the presence of other DSM-5 diagnoses, including bipolar disorder, schizophrenia, personality disorders, obsessive-compulsive disorder, intellectual disability, or panic disorder; (2) met the DSM-5 criteria for substance or alcohol abuse or dependence; (3) received physical therapies such as acupuncture, transcranial magnetic stimulation, or electroconvulsive therapy within the past 6 months; (4) history of brain surgery, trauma, impaired consciousness, epilepsy, febrile convulsions, or coma; (5) severe organic physical diseases, such as hypertension, cerebral parenchymal lesions, or cardiovascular diseases; (6) pregnant, lactating, or planning to conceive; (7) inability to comply with the prescribed treatment protocol; (8) abnormal coagulation function; or (9) modification of the primary diagnosis during hospitalization (e.g., diagnosis revised to bipolar disorder or schizophrenia).

Dropout criteria included: (1) withdrawal of consent or refusal to continue treatment after enrollment for any reason; (2) occurrence of intolerable or serious adverse events (e.g., palpitations, vomiting, dizziness) or safety incidents (e.g., broken needle, acupuncture fainting, or seizures) that prevented further treatment; (3) poor compliance or failure to cooperate during the treatment period; or (4) changes in pharmacological treatment, including the use of a single type of SSRI for ≤7 days, discontinuing SSRIs within 7 days, switching to another antidepressant, or combining treatments with other antidepressants.

### 2.3 Sample Size Calculation

Our preliminary analysis revealed a 47.2% ± 25.3% HAMD score reduction in the intervention group and 26.0% ± 30.4% reduction in the controls. Using PASS 2021 software (NCSS, LLC, Kaysville, UT, USA) with a 3:2 allocation ratio (α = 0.05, two-tailed, power = 0.8, superiority margin = 10%), the minimum required sample size was 170 participants (102 in the intervention group and 68 in the control group). After accounting for a predicted dropout rate of 20%, the initial recruitment target was set at 213 participants.

### 2.4 Randomization 

Participants were randomly assigned in a 3:2 ratio to the acupuncture group (n = 128) or the control group (n = 85) using SPSS Statistics 24.0 (IBM Corp) (Armonk, NY, USA), with simple randomization and no stratification or blocking. Treatment codes (1 or 2) were assigned at random. An independent researcher who was not involved in the treatment or assessments, conducted the allocation and generated the corresponding acupuncture prescriptions.

### 2.5 Treatment Plan

#### 2.5.1 Pharmacotherapy

All participants received standardized SSRI treatment. Dosage was titrated to clinical targets within two weeks and converted to fluoxetine equivalents for analysis [29,30]. All participants received routine SSRI treatment, and acupuncture was delivered by trained clinicians. Converted medications included escitalopram, sertraline, citalopram, paroxetine, and fluvoxamine.

#### 2.5.2 Acupuncture Protocol

In addition to medication, participants in the acupuncture group received the acupuncture protocol. The pre-treatment assessment was conducted 1 or 2 days before receiving the treatment. Treatment was administered once daily for five consecutive days, followed by a two-day break, comprising one treatment cycle. Over a two-week period, participants completed two such cycles, totaling 10 sessions (one session every 24 h). Each acupuncture session lasted 30 minutes. The post-treatment assessment was conducted either on the same day or the day following completion of the two-week treatment (**Supplementary Fig. 1**). Acupoint selection in the present study was guided by the theoretical framework and clinical principles described in a nationally recognized Traditional Chinese Medicine textbook [31,32]. This included Baihui (GV20), Dazhui (GV14), Fengfu (GV16), Fengchi (GB20), Neiguan (PC6), Shenmen (HT7), Sanyinjiao (SP6), Taixi (KI3), Taichong (LR3). More details about acupoints and acupuncture procedures can be found in **Supplementary Material 1**.

All needles were manipulated with even twirling to elicit the *deqi* sensation and retained for 30 minutes per session.

This study followed a standardized protocol. All practitioners were licensed acupuncturists with at least 11 years of TCM education and three years of clinical experience. Consistency training was conducted by two senior acupuncturists, comprising 8 or more rounds over a six-month period.

### 2.6 Clinical Assessment

The primary outcome of the study was the reduction rate in HAMD scores, which allows for better adjustment based on baseline severity [33]. Secondary outcomes included changes in cognitive function, anxiety levels, sleep quality (as measured by the Pittsburgh Sleep Quality Index), and suicidal ideation. The absolute change in the 17-item Hamilton Depression Rating Scale (HAMD-17) [34] was included as a secondary outcome to supplement the primary analysis.

Cognitive function was evaluated using the Mini-Mental State Examination (MMSE) [35]. This has a maximum score of 30, with scores ≤27 indicating potential cognitive impairment. Anxiety was assessed with the Hamilton Anxiety Rating Scale (HAMA) [36], where scores ≤7 suggest no anxiety, and scores ≥14 indicate the presence of anxiety symptoms. Sleep quality over the preceding month was evaluated using the Pittsburgh Sleep Quality Index (PSQI) [37]. A total score of 5 or less indicates good sleep quality, while scores ≥6 reflect varying levels of sleep disturbance. Suicidal ideation was assessed using the Beck Scale for Suicide Ideation (BSI) [38], with a score ≥6 indicating the presence of suicidal thoughts.

All scale assessments were repeated after two weeks of treatment to evaluate the effectiveness of acupuncture. The scales were administered by psychiatrists or graduate-level researchers who received professional training. All assessors participated in at least 8 rounds of a three-month standardized training program and achieved an inter-rater consistency of ≥80%. The trial was completed as planned.

### 2.7 Statistical Analysis

Data management was conducted using EpiData version 3.1 (EpiData Association, Odense, Denmark), with all data subjected to double-entry and cross-validation to ensure accuracy. Statistical analyses were performed using SPSS version 24.0. Primary analyses were based on the intention-to-treat (ITT) principle. To handle missing data under the missing-at-random (MAR) assumption, multiple imputation (MI) with 5 iterations was performed. As a sensitivity analysis, a per-protocol (PP) analysis was also conducted, restricting the sample to participants who fully completed the treatment and post-treatment assessments. All analyses were performed based on the ITT population (n = 213), with missing data handled using multiple imputation.

Continuous variables are presented as Mean ± standard deviation (SD), and categorical variables as frequencies and percentages. The Kolmogorov-Smirnov test was employed to assess the normality of data distribution. Baseline demographic and clinical characteristics were compared between groups using independent *t*-tests or Mann-Whitney U tests for continuous variables, and chi-square tests for categorical variables. To rigorously evaluate the efficacy of adjunctive acupuncture while controlling for baseline imbalances (e.g., years of education and baseline scores), Analysis of Covariance (ANCOVA) was performed on post-treatment scores, with their respective baseline values and years of education included as covariates. For within-group pre- and post-treatment comparisons, paired *t*-tests or Wilcoxon signed-rank tests were used. Correlations between change in Hamilton Depression Rating Scale scores (ΔHAMD) and baseline clinical/demographic variables (including baseline HAMD, HAMA, PSQI, MMSE, BSI, gender, age, education, age at first onset, illness duration, and fluoxetine-equivalent dose) were analyzed using Spearman’s correlation coefficients.

To identify clinical predictors of antidepressant efficacy within the acupuncture group (n = 128), we conducted hierarchical linear regression analysis with ΔHAMD as the dependent variable. In Model 1, years of education and baseline BSI scores were entered as nuisance covariates to control for baseline imbalances. In Model 2, baseline clinical characteristics (HAMD, HAMA, PSQI, and illness duration) were added to evaluate their incremental predictive value. Multicollinearity was assessed using the Variance Inflation Factor (VIF) diagnostics.

To control the false discovery rate in multiple testing, *p*-values were adjusted using the Benjamini-Hochberg (FDR) correction. A two-sided adjusted *p*-value < 0.05 was considered statistically significant.

## 3. Results

### 3.1 Socio-Demographic and Baseline Clinical Characteristics

Socio-demographic and baseline clinical data are summarized in Table 1. Independent *t*-tests or Kolmogorov-Smirnov (K-S) tests were employed for continuous variables based on their distribution normality, while the Chi-square test was used for categorical data. No significant differences were observed between the two groups in terms of age (*t* = 1.405, *p* = 0.162), gender (*χ^2^* = 0.160, *p* = 0.692), age at first onset (*t* = 1.382, *p *= 0.169), total duration of illness (*t* = 0.197, *p* = 0.844), or fluoxetine equivalent dosage (*t* = –0.771, *p* = 0.442).

**Table 1.** **Socio-demographic profile of the acupuncture and control groups at baseline**.

	Acupuncture (n = 128)	Control (n = 85)	*t/Z/χ* ^2^	*p*
Gender (M/F)	34/94	25/60	0.160^a^	0.692
Age (years)	25.83 ± 12.21	23.22 ± 12.68	1.405^b^	0.162
Education (years)	12.40 ± 3.25	11.44 ± 3.25	1.979^b^	0.049*
Age at first onset (years)	22.13 ± 11.36	19.80 ± 11.16	1.382^b^	0.169
Total duration of illness (months)	42.68 ± 44.21	41.30 ± 50.52	0.197^b^	0.844
N0PSQI	13.28 ± 3.81	14.18 ± 3.32	–1.658^b^	0.099
N0BSI	22.82 ± 5.94	24.82 ± 4.56	–2.608^b^	0.010*
N0MMSE	26.64 ± 3.74	26.08 ± 2.26	1.262^b^	0.209
N0HAMD	26.58 ± 5.49	28.06 ± 5.30	0.851^c^	0.464
N0HAMA	23.66 ± 6.62	24.29 ± 5.88	0.862^c^	0.447
Fluoxetine equivalent (mg)	24.46 ± 26.91	28.53 ± 45.11	–0.771^b^	0.442

**Notes**: ^a^ Chi-square value; ^b^ *t*-test value; ^c^ K-S test-value. M, Male; F, Female; N0PSQI, Baseline Pittsburgh Sleep Quality Index; N0BSI, Baseline Beck Scale for Suicide Ideation; N0MMSE, Baseline Mini-Mental State Examination; N0HAMD, Baseline Hamilton Depression Rating Scale; N0HAMA, Baseline Hamilton Anxiety Rating Scale; K-S, Kolmogorov-Smirnov; *, *p* < 0.05.

Regarding baseline clinical assessments, no significant group differences were found for N0PSQI (*p* = 0.099), N0MMSE (*p* = 0.209), N0HAMD (*p* = 0.464), or N0HAMA (*p* = 0.447). However, significant baseline imbalances were identified for years of education (*t* = 1.979, *p* = 0.049) and BSI scores (*t* = –2.608, *p* = 0.010). To ensure the validity of the efficacy comparisons, these unbalanced baseline variables were subsequently incorporated as covariates in the Analysis of Covariance (ANCOVA) models to control for potential confounding effects on treatment outcomes.

### 3.2 Baseline and Concomitant Medication

At baseline, there were no significant differences between the two groups in terms of the percentage of participants receiving antipsychotics (*χ*^2^ = 3.873,* p *= 0.062), mood stabilizers (*χ*^2^ = 0.908, *p* = 0.512), benzodiazepines (*χ*^2^ = 2.279, *p* = 0.108), or antidepressants (*χ*^2^ = 2.656, *p* = 0.142). Furthermore, no significant difference was observed in the initial antidepressant dosage (calculated as fluoxetine equivalents) between the two groups (*p* > 0.05), indicating high comparability in their baseline pharmacological status.

Following enrollment, participants in both the combined treatment and pharmacotherapy alone groups were prescribed a single SSRI by clinicians based on clinical judgment. No participants were permitted to combine multiple antidepressants, and those who discontinued or switched antidepressants during the study were excluded from the final analysis. At the two-week follow-up, no significant differences were found in the concurrent use of antipsychotics (*χ*^2^ = 0.079, *p* = 0.778), mood stabilizers (*χ*^2^ = 0.049, *p *= 0.835), benzodiazepines (*χ*^2^ = 0.009, *p* = 0.923), or the types of SSRIs prescribed (*χ*^2^ = 1.205, *p* = 0.932).

A statistical difference was observed in the fluoxetine-equivalent dosage at follow-up (*p* < 0.05), with the combined treatment group receiving 35 (20, 44) mg and the pharmacotherapy alone group receiving 40 (28, 44) mg. However, this variation was unlikely to have clinical significance. Previous evidence from randomized controlled trials suggests that there is no clear dose–response relationship for SSRIs within the commonly used therapeutic dose range, and increasing the dose beyond the standard range does not consistently result in superior efficacy for major depressive disorder (MDD) [30]. Therefore, the pharmacological intervention remained comparable between the two groups throughout the study.

### 3.3 Comparative Efficacy of Acupuncture Plus Medication Versus Medication Alone in Symptom Reduction

To rigorously evaluate the comparative efficacy of adjunctive acupuncture, ANCOVA was performed on post-treatment scores, using respective baseline scores and years of education as covariates to control for potential confounding factors. As shown in Table 2, the acupuncture group demonstrated significantly greater symptomatic reduction compared to the control group across all dimensions. Specifically, significant between-group differences were observed in post-treatment HAMD (*F* = 11.337, *p* < 0.001, partial η^2^ = 0.058) and HAMA scores (*F* = 11.634, *p* < 0.001, partial η^2^ = 0.060).

**Table 2.** **ANCOVA results of clinical scale scores between the acupuncture and control groups**.

	Acupuncture (Mean ± SD)	Control (Mean ± SD)	*F*	partial η^2^	*p*	*q-value*
N1HAMD	12.57 ± 8.83	17.31 ± 6.66	11.337	0.058	<0.001	<0.001
N1HAMA	11.47 ± 6.70	15.28 ± 7.22	11.634	0.060	<0.001	<0.001
N1PSQI	8.61 ± 3.06	10.40 ± 3.13	12.060	0.062	<0.001	<0.001
N1MMSE	27.59 ± 2.08	26.37 ± 3.69	5.393	0.028	0.021	0.021

**Notes**: Analysis of Covariance (ANCOVA) was performed to compare clinical scores between the acupuncture group and the control group at Week 2, using baseline scores, years of education, and baseline BSI scores as covariates to adjust for potential confounding effects; N1HAMD, Hamilton Depression Rating Scale at Week 2; N1HAMA, Hamilton Anxiety Rating Scale at Week 2; N1PSQI, Pittsburgh Sleep Quality Index at Week 2; N1MMSE, Mini-Mental State Examination at Week 2; SD, standard deviation; partial η^2^ represents the effect size of the treatment; *q-value* represents the False Discovery Rate (FDR)-corrected *p*-values for multiple comparisons. *p* < 0.05 or *q* < 0.05 was considered statistically significant.

Notably, after adjusting for baseline imbalances, the acupuncture group also showed significantly better outcomes in sleep quality (PSQI: *F* = 12.060, *p* < 0.001) and cognitive function (MMSE: *F* = 5.393, *p *= 0.021) compared to the medication-only group. All primary and secondary outcomes remained statistically significant after False Discovery Rate (FDR) correction (*q* < 0.05). These results indicate that acupuncture combined with medication provides a more comprehensive therapeutic benefit for both core depressive symptoms and common comorbidities than medication alone (Table 2).

### 3.4 Baseline Predictors of Antidepressant Response to Combined Acupuncture Treatment

**Fig. 2.**
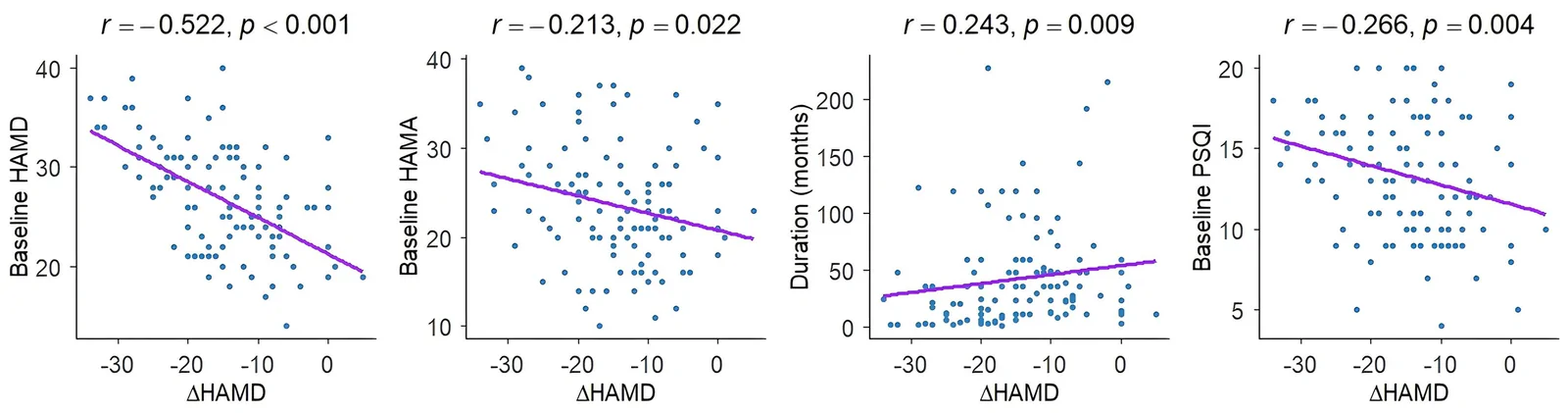
**Correlations of baseline depression and anxiety severity, and duration with clinical outcomes in the acupuncture group**. Scatter plots depicting correlations between baseline HAMD, baseline HAMA, duration of illness, and baseline PSQI scores with ΔHAMD. Significant negative correlations were observed for HAMD, PSQI and HAMA, while a positive correlation was noted for duration. ΔHAMD represents the change in HAMD scores. Statistical significance was set at *p* < 0.05.

Having confirmed significant treatment differences, we analyzed correlations between ΔHAMD and baseline HAMD, baseline HAMA, baseline PSQI, baseline MMSE, baseline BSI, gender, age, education, age at first onset, Fluoxetine equivalent, and duration of illness. Significant negative correlations were observed between ΔHAMD and baseline HAMD (r = –0.522,* p *< 0.001), baseline PSQI (r = –0.266, *p* = 0.004), and baseline HAMA (r = –0.213,* p *= 0.022), while a positive correlation was found with illness duration (r = 0.243, *p* = 0.009). No correlation was apparent between ΔHAMD and baseline BSI (r = –0.147, *p* = 0.117) (Fig. 2).

To identify clinical predictors of antidepressant efficacy, a hierarchical linear regression analysis was performed within the acupuncture group (n = 128), using ΔHAMD as the dependent variable (Table 3).

**Table 3.** **Hierarchical regression analysis for predictors of ΔHAMD**.

Model	Predictor	*B*	*β*	*R^2^*	*ΔR^2^*	*p*	*q-value*
Model 1	Education (years)	–0.206	–0.068	0.022	0.022	0.476	-
	N0BSI	–0.238	–0.143	0.022	0.022	0.135	-
Model 2	Education (years)	–0.129	–0.042	0.287	0.265	0.631	0.757
	N0BSI	0.099	0.059	0.287	0.265	0.513	0.757
	N0PSQI	–0.033	–0.013	0.287	0.265	0.895	0.895
	N0HAMD	–1.336	–0.741	0.287	0.265	<0.001*	0.006*
	N0HAMA	0.574	0.384	0.287	0.265	0.002*	0.006*
	Duration of illness	0.010	0.045	0.287	0.265	0.617	0.757

**Notes**: N0HAMD, Baseline Hamilton Depression Rating Scale; N0HAMA, Baseline Hamilton Anxiety Rating Scale; N0BSI, Baseline Beck Scale for Suicide Ideation; N0PSQI, Baseline Pittsburgh Sleep Quality Index; *B*, unstandardized coefficient; *R^2^*, Proportion of variance explained by the model; *ΔR^2^*, Change in *R^2^* between Model 1 and Model 2; *β*, standardized coefficient; Model 1, *F* = 1.261, *p* = 0.288; Model 2, *F* = 7.183, *p* < 0.001; *q-value* represents the False Discovery Rate (FDR)-corrected *p*-values for multiple comparisons; *, *p* < 0.05.

In Model 1, years of education and baseline BSI scores were entered as nuisance covariates to control for potential confounding effects caused by baseline imbalances. This initial model did not significantly predict treatment outcomes (*R^2^* = 0.022, *p* = 0.287).

In Model 2, baseline clinical characteristics—including HAMD, HAMA, PSQI scores, and total duration of illness—were added to the model. The inclusion of these clinical variables significantly enhanced the model’s predictive power (*ΔR^2^* = 0.265, *p* < 0.001), with the total model explaining 28.7% of the variance in ΔHAMD.

Specifically, baseline HAMD scores and baseline HAMA scores emerged as the only two independent predictors of symptomatic improvement. Each 1-point increase in the baseline HAMD score was associated with a 1.336-point decrease in ΔHAMD (*B* = –1.336, *β* = –0.741, *p* < 0.001), suggesting that patients with higher initial depression severity experienced relatively less symptom reduction during the two-week intervention. Conversely, the baseline HAMA score was a positive predictor, with each 1-point increase corresponding to a 0.574-point increase in ΔHAMD (*B* = 0.574, *β* = 0.384, *p *= 0.002).

Other factors, including years of education, baseline BSI, baseline PSQI, and total duration of illness, did not significantly contribute to the regression model (all *p* > 0.05). These results indicate that baseline depression and anxiety levels are the most robust indicators of early treatment response to adjunctive acupuncture.

### 3.5 Safety Analysis

No serious adverse events, such as broken needles or subcutaneous hematoma, occurred during the study. Additionally, no severe adverse reactions, including hypotensive shock or seizures, were observed. The incidence of adverse effects—such as dry mouth, nausea, vomiting, and constipation—was significantly lower in the combined treatment group compared to the pharmacotherapy alone group (refer to **Supplementary Material 2** for detailed data).

## 4. Discussion

To our knowledge, this is the first clinical study to evaluate the efficacy of the acupuncture technique and to compare its effectiveness, when combined with SSRIs, to that of antidepressant treatment alone in patients with depression and comorbid symptoms. The main findings of this study can be summarized as follows: (1) both combination therapy and pharmacotherapy alone proved effective in treating depression and comorbid symptoms, including anxiety, cognitive impairment, sleep disturbances, and suicidal ideation; (2) the results of between-group analysis indicated that integrated therapy exhibited greater efficacy than monotherapy in improving depression and its prevalent comorbid symptoms, such as anxiety, cognitive dysfunction, and sleep disturbances; and (3) after controlling for sociodemographic variables, linear regression analysis identified baseline HAMD scores as a risk factor and baseline HAMA scores as a protective factor for treatment efficacy in the acupuncture group.

This study found that both treatment modalities proved effective for alleviating depressive symptoms and associated comorbidities, in line with findings from previous research. However, our results demonstrated greater improvement in the acupuncture group, not only in core depressive symptoms but also in prevalent comorbidities, including anxiety, sleep disturbances, and cognitive impairment, thus supporting the role of acupuncture as an effective adjunctive treatment. Prior RCTs comparing electroacupuncture and SSRIs reported greater improvements in anxiety, hopelessness, and Clinical Global Impression (CGI) scores in electroacupuncture-treated groups [39]. A multicenter RCT involving 477 patients with moderate-to-severe depression found that combining acupuncture with SSRIs improved HAMD response rates, accelerated the onset of response, and improved SDS and CGI scores compared with SSRIs alone [4]. Other studies have supported the use of acupuncture in both anxious depression (AD) and non-anxious depression (NAD), showing symptom improvements and faster SSRI response in NAD cases [40]. In university students, acupuncture combined with moxibustion was reported to be more effective and faster-acting than oral fluoxetine, with fewer adverse effects [41]. These results suggest that acupuncture may exert its therapeutic effects by modulating the reward and motivation circuits within the cortico-striatal system in patients with MDD [17], thereby improving emotional feedback and leading to clinical recovery. Although no previous studies have specifically compared acupuncture versus medication for the treatment of depression with comorbid anxiety, both acupuncture and electroacupuncture have demonstrated efficacy in treating anxiety disorders as monotherapy or adjunctive therapy [23]. A double-blind RCT involving 112 patients with anxiety disorders found that SSRIs combined with real acupuncture achieved significantly greater reductions in Spielberger State-Trait Anxiety Inventory (STAI) scores than SSRIs alone or SSRIs plus sham acupuncture [42]. A study of chronic insomnia with comorbid depression and anxiety found that acupuncture significantly reduced HAMD and HAMA scores, decreased serum corticosterone, and increased 5-HT levels, suggesting that biomarker modulation may be a potential mechanism [43].

Beyond mood regulation, the combined treatment group exhibited superior performance in terms of improving sleep quality and cognitive function compared to the pharmacotherapy alone group, in agreement with previous research findings. Prior studies have suggested that insomnia symptoms in depression are associated with dysregulation of the hypothalamic–pituitary–adrenal (HPA) axis, resulting in alterations in cortisol (CORT) secretion and other neurobiological processes, including serotonergic neurotransmission [44,45]. Traditional acupuncture has been demonstrated to alleviate insomnia and depressive symptoms by modulating neurotransmitter and serum hormone levels [46]. This further establishes acupuncture as a viable adjunctive intervention for insomnia, particularly given the high prevalence of residual sleep disturbances reported in MDD patients following pharmacotherapy or cognitive behavioral therapy [47,48]. Neuroimaging evidence further suggests that stimulation of specific acupoints, such as Baihui (GV 20), can trigger changes in cognitive-related brain activity, including enhanced functional connectivity and neurotransmitter alterations [49,50]. In summary, our findings suggest that acupuncture possesses distinct advantages in managing the “symptom clusters” of depression. Additionally, our results indicated that higher baseline HAMD scores predicted poorer outcomes, whereas higher baseline HAMA scores were associated with better responses to acupuncture. Recent studies have investigated the antidepressant effects of acupuncture compared with placebo controls, although further evidence from completed clinical trials is still needed [51]. For instance, a study by Wang et al. [52] found that optimized acupuncture protocols demonstrated superiority over standard protocols only in mildly symptomatic subgroups of patients with depressive insomnia, as measured by HAMD scores. A recent meta-analysis reported that acupuncture moderately reduces the severity of MDD, with combination therapy being more effective than acupuncture alone [20]. This implies that acupuncture may be insufficient as monotherapy for severe depression but can serve as an effective adjunct to accelerate response and reduce adverse effects. In milder cases, acupuncture may be more adaptable and cost-effective. High baseline HAMD scores may reflect severe symptomatology and contribute to poorer outcomes with acupuncture alone. A dose-response study indicated that 66% of MDD patients required up to 36 sessions to achieve maximal improvement of HAMD scores, suggesting that patients with more severe depression need more treatment sessions [53]. Furthermore, individuals with severe symptoms may be more likely to discontinue treatment prematurely due to comorbidities or psychosocial barriers. These factors may explain the reduced efficacy observed in patients with higher baseline HAMD scores. Conversely, a meta-analysis on anxiety disorders found that acupuncture significantly improved HAMA scores, total response rates, and SAS scores compared to controls [54]. Moreover, a placebo-controlled trial in patients with generalized anxiety disorder reported an 85.7% response rate with real acupuncture [55]. These findings support the hypothesis that more severe anxiety symptoms may predict greater responsiveness to acupuncture. The basis for this clinical observation may be related to the distinct neurobiological profiles associated with anxiety severity in depression. Recent evidence from the BIGHI cohort has identified significant correlations between cognitive dysfunction and altered amygdala functional connectivity in treatment-naïve patients with depression and comorbid anxiety [56]. Furthermore, research on EEG microstate dynamics has revealed that increasing anxiety severity in MDD is associated with progressive dysfunction in large-scale brain networks, particularly involving the sensorimotor network [57]. These shared and specific neurobiological signatures highlight the complexity of depressive symptoms with comorbid anxiety and may provide a mechanistic basis for the differential treatment responses observed in the current study.

### Limitations

This study has several limitations. First, all outcome measures were subjective scales and lacked objective or biomarker-based endpoints. Second, the follow-up period was limited to 14 days post-treatment, preventing the evaluation of long-term efficacy. Third, slight group differences in sample size and baseline characteristics could not be fully controlled. Finally, this study did not include a sham acupuncture control; therefore, the specific contribution of acupuncture-related effects beyond non-specific factors could not be fully determined. However, this study aimed to evaluate the additional clinical benefit of acupuncture when combined with standard pharmacotherapy compared with SSRI treatment alone. Further studies with longer follow-up periods and objective outcome measures are needed to confirm these results.

## 5. Conclusions

In summary, both acupuncture and antidepressant medication proved effective for alleviating depressive symptoms and related comorbidities. However, combination therapy offers a rapid and comprehensive therapeutic advantage for inpatients with MDD. This combined approach significantly outperformed medication alone in reducing the severity of depression, anxiety, insomnia, and cognitive dysfunction over a two-week period. These results suggest that acupuncture is a potent adjunctive strategy for treating the complex symptom presentations of depression.

## Data Availability

The data underlying this study are available upon reasonable request from the corresponding authors. Deidentified participant data, the study protocol, and statistical analysis code will be made available for academic use upon request and subject to institutional approval.
