# Supplementary material for: Efficacy of Acupuncture Combined With Pharmacotherapy for Depression: A Randomized Controlled Clinical Trial
Source: Alpha Psychiatry. 2026 Aug 13;27(4):52350. doi: 10.31083/AP52350 (PMC13539995; doi:10.31083/AP52350)
Supplement: Supplementary file 1 [file 2757-8038-27-4-52350-s1.zip › Supplementary Material/Supplementary Fig 1.docx]

**Supplementary Fig. 1. Timeline of the acupuncture intervention and assessment schedule.** Participants received acupuncture treatment once daily for 5 consecutive days per week over a 2-week period. Each acupuncture session lasted 30 minutes. The intervention consisted of two treatment cycles (Days 1–5 and Days 8–12), with rest periods on Days 6–7 and Days 13–14. Baseline assessment was performed before treatment initiation, and post-treatment assessment was conducted on Day 13 or 14.
