# Supplementary material for: Efficacy of Acupuncture Combined With Pharmacotherapy for Depression: A Randomized Controlled Clinical Trial
Source: Alpha Psychiatry. 2026 Aug 13;27(4):52350. doi: 10.31083/AP52350 (PMC13539995; doi:10.31083/AP52350)
Supplement: Supplementary file 1 [file 2757-8038-27-4-52350-s1.zip › Supplementary Material/Supplementary Material 1.docx]

**Intervention**

**1. Pharmacotherapy (Control Group)**

Participants in the control group received monotherapy with a Selective Serotonin Reuptake Inhibitor (SSRI). The specific SSRI was selected and titrated to the target clinical dose within two weeks based on the patient’s clinical response and tolerability (Sertraline 50–200 mg/d; Escitalopram 10–20 mg/d; Citalopram 20–40 mg/d; Fluoxetine 20–60 mg/d; Fluvoxamine 100–300 mg/d; or Paroxetine 20–50 mg/d). When clinically necessary, combined use of antipsychotics, mood stabilizers, or benzodiazepines was permitted. To ensure data validity, participants who maintained a consistent single SSRI regimen for more than 7 days were included in the final follow-up and analysis according to the predefined study protocol.

**2. Combined Treatment (Acupuncture + SSRI Group)**

The acupuncture treatment in this study was based on the “Tongyang Xingshen” acupuncture protocol developed by Professor **Wei Han** from the Affiliated Brain Hospital of Guangzhou Medical University. The theoretical principles of this method are detailed in *Chinese Acupuncture & Moxibustion*, April 2023, Vol. 43, No. 4 (DOI: 10.13703/j.0255-2930.20221129-k0003).

The specific acupoint selection and manipulation procedures used in this study were as follows:

In addition to medication, participants in the acupuncture group received the "Tongyang Xingshen" acupuncture protocol. Outcome assessments were conducted either on the same day or the day following the completion of the two-week treatment. Acupoint selection followed guidelines in the national TCM education textbook published by the People's Medical Publishing House[1, 2].

**2.1 Acupuncture Apparatus**

All acupuncture procedures were performed using sterile, single-use disposable needles of the **Hwato brand** (manufactured by Suzhou Medical Supplies Factory Co., Ltd., Jiangsu, China). Two specific sizes of needles were utilized depending on the anatomical location of the acupoints: 0.30 mm × 25 mm, 0.30 mm × 40 mm.

**2.2 Preparation and Position**

Prior to the procedure, participants were instructed to empty their bladders. Depending on the targeted acupoints and clinical requirements, participants were placed in either a supine or prone position. The skin at and around the selected acupoint sites was strictly disinfected using 75% isopropyl alcohol before needle insertion.

**2.3 Detailed Acupoint Selection and Manipulation Techniques**

The "Tongyang Xingshen" (Promoting Yang and Awakening the Spirit) protocol involved a specific sequence and precise needle manipulation to ensure the induction of the "Deqi" sensation (a characteristic feeling of soreness, numbness, or heaviness).

| Acupoint (Code) | Localization | Insertion Technique & Depth | Sensation/Requirement |
| --- | --- | --- | --- |
| Baihui (GV20) | At the vertex, on the midline. | **First needle.** Flat insertion (15–20 mm) anteriorly, penetrating the sub-aponeurotic layer. | Feeling of heaviness or distension. |
| Yintang (GV29) | Midpoint between the eyebrows. | The skin was pinched and the needle inserted inferiorly (flat insertion, 7–12 mm). | Local soreness. |
| Fengfu (GV16) | Below the external occipital protuberance. | Inserted 13–20 mm toward the tip of the nose. | Careful depth control. |
| Dazhui (GV14) | Below the spinous process of C7. | Oblique insertion upward (45° angle) to a depth of 13–20 mm. | Deqi sensation. |
| Taodao (GV13) | Below the spinous process of T1. |  |  |
| Shenzhu (GV12) | Below the spinous process of T3. |  |  |
| Shendao (GV11) | Below the spinous process of T5. |  |  |
| Zhiyang (GV9) | Below the spinous process of T7. |  |  |
| Jinsuo (GV8) | Below the spinous process of T9. |  |  |
| Lieque (LU7) | Proximal to the styloid process. |  |  |

Note: All needles were manipulated using an even twirling method (uniform rotation) until the Deqi sensation was achieved. The needles were retained in situ for a total of 30 minutes per session.

**2.4 Intervention Schedule and Inclusion Rationale**

**Dosage:** One session daily, five consecutive days per week (Monday to Friday), followed by a two-day break.

**Total Sessions:** 10 sessions completed within a 2-week period.

**Inclusion for Analysis:** In accordance with the predefined study protocol, participants who completed at least 5 acupuncture sessions within the 2-week treatment window were considered to have received adequate treatment exposure and were included in the final analysis.

**2.5 Practitioner Qualifications and Quality Control**

To ensure the high internal validity of the intervention:

**1. Clinical Experience:** All treatments were administered by licensed TCM practitioners with a minimum of eleven years of academic TCM education and at least three years of specialized clinical experience in acupuncture.

**2. Consistency Training:** Before the trial commenced, all practitioners underwent rigorous standardization training. This training was supervised by two senior acupuncturists and consisted of eight or more rounds of technical evaluation over a six-month period to ensure uniform needle depth, angle, and manipulation across all participants.

This study followed a standardized protocol. All practitioners were licensed acupuncturists with at least eleven years of TCM education and three year of clinical experience. Consistency training was conducted by two senior acupuncturists, spanning more than a six-month period.

[1] Jia C. Traditional Chinese Medicine. Beijing: People's Medical Publishing House.

[2] Hu L, Liu Q. Meridian and Acupoint Theory, 2nd ed. Shanghai Science and Technology Press: 2018.
