# Supplementary material for: Efficacy of Acupuncture Combined With Pharmacotherapy for Depression: A Randomized Controlled Clinical Trial
Source: Alpha Psychiatry. 2026 Aug 13;27(4):52350. doi: 10.31083/AP52350 (PMC13539995; doi:10.31083/AP52350)
Supplement: Supplementary file 1 [file 2757-8038-27-4-52350-s1.zip › Supplementary Material/Supplementary Material 2.docx]

| **The names of adverse effects** | **Percentage (%)** |
| --- | --- |
| Tremor | 3.5 |
| Dry mouth | 7.0 |
| Nasal congestion | 2.6 |
| Constipation | 4.3 |
| Sweating | 0.9 |
| Nausea and vomiting | 3.5 |
| Diarrhea | 3.5 |
| Dizziness and syncope | 1.7 |
| Tachycardia | 1.7 |
| Weight gain | 4.3 |
| Headache | 0.9 |
| Others | 2.6 |
| Total | 21.7 |

Acupuncture Group：

Control Group:

| **The names of adverse effects** | **Percentage (%)** |
| --- | --- |
| Excitement or agitation | 2.8 |
| Dysphoria | 1.4 |
| Increased activity | 1.4 |
| Somnolence | 4.1 |
| Tremor | 6.9 |
| Akathisia | 4.1 |
| Dry mouth | 27.4 |
| Blurred vision | 1.4 |
| Constipation | 13.7 |
| Sweating | 1.4 |
| Nausea and vomiting | 31.5 |
| Diarrhea | 1.4 |
| Dizziness and syncope | 6.8 |
| Skin symptoms | 2.8 |
| Weight gain | 4.2 |
| Weight loss | 2.7 |
| Anorexia | 4.1 |
| Others | 2.7 |
| Total | 55.4 |
